# Positioning food standards programmes to protect public health: current performance, future opportunities and necessary reforms

**DOI:** 10.1017/S1368980018003786

**Published:** 2019-04

**Authors:** Mark Andrew Lawrence, Christina Mary Pollard, Tarun Stephen Weeramanthri

**Affiliations:** 1 Institute for Physical Activity and Nutrition (IPAN), Deakin University, 1 Gheringhap Street, Geelong, VIC 3220, Australia; 2 School of Public Health, Curtin University, Perth, Western Australia, Australia; 3 School of Population and Global Health, University of Western Australia, Perth, Western Australia, Australia

**Keywords:** Food standards programme, Food regulation system, Nutrient profiling, Food policy, Risk analysis, Food Standards Australia New Zealand

## Abstract

**Objective:**

To assess current performance and identify opportunities and reforms necessary for positioning a food standards programme to help protect public health against dietary risk factors.

**Design:**

A case study design in which a food standards programme’s public health protection performance was analysed against an adapted Donabedian model for assessing health-care quality. The criteria were the food standards programme’s structure (governance arrangements and membership of its decision-making committees), process (decision-making tools, public engagement and transparency) and food standards outcomes, which provided the information base on which performance quality was inferred.

**Setting:**

The Australia and New Zealand food standards programme.

**Participants:**

The structure, process and outcomes of the Programme.

**Results:**

The Programme’s structure and processes produce food standards outcomes that perform well in protecting public health from risks associated with nutrient intake excess or inadequacy. The Programme performs less well in protecting public health from the proliferation and marketing of ‘discretionary’ foods that can exacerbate dietary risks. Opportunities to set food standards to help protect public health against dietary risks are identified.

**Conclusions:**

The structures and decision-making processes used in food standards programmes need to be reformed so they are fit for purpose for helping combat dietary risks caused by dietary excess and imbalances. Priorities include reforming the risk analysis framework, including the nutrient profiling scoring criterion, by extending their nutrition science orientation from a nutrient (reductionist) paradigm to be more inclusive of a food/diet (holistic) paradigm.

A food standards programme is a powerful setting for protecting public health^(^ ^1^ ^–^ ^3^ ^)^. Food standards can influence both the supply (composition standards) and the demand (food labelling standards) for food. Globally, the Joint FAO/WHO Food Standards Programme is managed by the Codex Alimentarius Commission (Codex). Codex was established in 1963 to develop harmonised global food standards, guidelines and codes of practice, and provides opportunities for tackling public health problems. Article 1(a) of the Codex Statutes states that the Commission be responsible for, ‘all matters pertaining to the implementation of the Joint FAO/WHO Food Standards Programme, the purpose of which is: (a) protecting the health of the consumers and ensuring fair practices in the food trade …’^(^ ^4^ ^)^. National food standards programmes are broadly consistent with Codex procedures and typically their priority objective is to protect public health and safety in the setting of food standards.

Food standards programmes are complex settings for tackling public health problems^(^ ^5^ ^)^. They exist not only to protect public health and safety, but also to ensure fair food trade practices. These dual mandates often result in competing objectives and interests, which a food standards programme needs to contend with during decision making. Tensions arise in balancing food’s role as a health prerequisite as well as a commercial commodity central to the political economy of many countries. Inevitably, the decision-making activities around food standards programmes are political^(^ ^6^ ^,^ ^7^ ^)^. Tensions towards these activities can also arise within the public health community itself^(^ ^8^ ^)^. Often there is a lack of clarity regarding the interpretation of protecting public health and safety and how this will be applied – whether reacting to applications made to vary standards or proactively in setting standards to protect public health^(^ ^9^ ^)^.

Historically, food standards were introduced to protect the public from threats to food safety, fraud and adulteration. With dietary risk factors now the leading contributors to the global burden of disease, food standards programmes need to ensure they address dietary excesses and imbalances leading to obesity and diet-related non-communicable diseases (NCD)^(^ ^10^ ^)^. More than 100 countries have developed national food-based dietary guidelines to help tackle these dietary risk factors^(^ ^11^ ^)^. Guidelines recommend a dietary pattern emphasising a variety of nutritious whole foods and limiting energy-dense, nutrient-poor food products. However, there is a significant gap between recommended and actual dietary behaviour in many countries. For example, in Australia, a large body of evidence derived from multiple sources has been synthesised to inform the classification of nutritious foods into each of five food groups as well as non-essential foods into a ‘discretionary’ food group. Yet, across all age and gender groups, the majority under-consume the recommended number of servings for all the ‘five food group’ foods while at least 35 % of adults’ and 39 % of children’s total energy intake is derived from discretionary (energy-dense, nutrient-poor) food products^(^ ^12^ ^)^.

The present research aimed to assess current performance and identify opportunities and reforms necessary for positioning a food standards programme to help protect public health against dietary risk factors. Food safety in relation to microbiological and toxicological risks, fraud and adulteration was outside the investigation’s scope. Food Standards Australia New Zealand (FSANZ) and its activities in supporting Australian Dietary Guidelines’ recommendations, i.e. promoting ‘five food group’ foods and discouraging discretionary foods, served as a case study for the investigation. In 2014, the FSANZ Board endorsed the statement that when setting food standards FSANZ ‘adopts an evidence-based approach that applies appropriate methodologies in assessing the short-term and long-term risks to public health and safety’^(^ ^13^ ^)^. The Australia New Zealand Ministerial Forum on Food Regulation (the ‘Forum’ hereafter) then articulated a proactive stance, identifying three priority areas for the food regulation system, including to ‘support public health objectives to reduce chronic disease related to overweight and obesity’ as one of three priority areas for the food regulatory system 2017–2021^(^ ^14^ ^)^.

## Method

### Research design

**Fig. 1:**
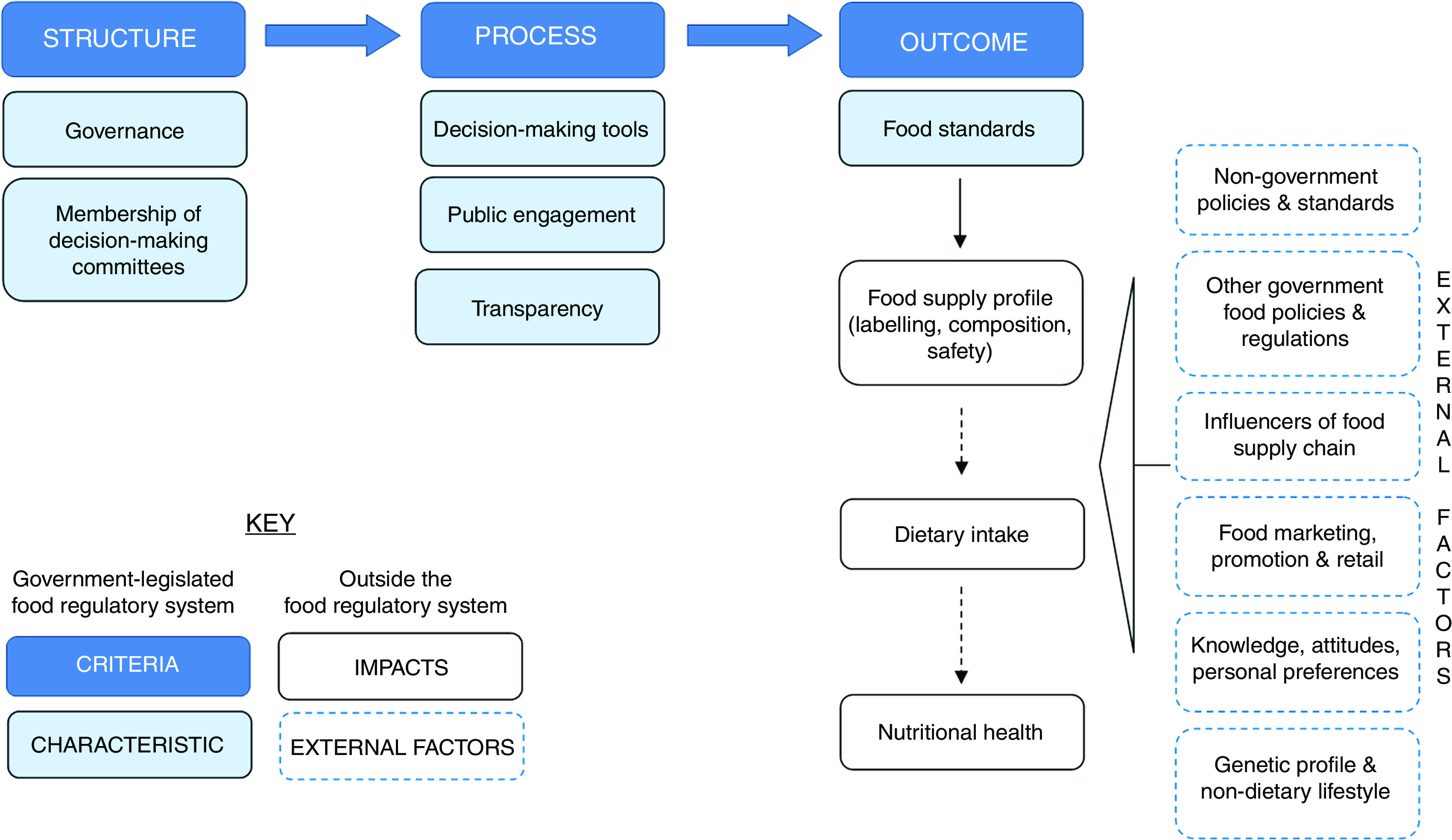
(colour online) Hypothetical relationships among characteristics of Donabedian’s structure, process and outcome criteria of performance in a food standards programme context

The Donabedian management model^(^ ^15^ ^)^ extensively used for defining and improving health-service quality was applied sequentially to assess the performance of the Australia and New Zealand (ANZ) food standards programme in relation to dietary risk factors for obesity and NCD. The Donabedian model employs a triad of criteria – structure, process and outcomes – to inform audits, assessments, evaluations and quality management^(^ ^16^ ^)^. The structure, process and outcomes are not attributes of performance, instead they are the information base on which one can infer performance quality. Structure influences process, and process influences outcome. The structure and process criteria of the food standards programme are themselves determined by overarching legislation, the FSANZ Act 1991^(^ ^17^ ^)^. Figure 1 outlines hypothetical relationships among characteristics of Donabedian’s structure, process and outcome criteria in the context of how well a food standards programme performs.

The model has a practical purpose. The assessments can be interrogated to identify opportunities and necessary reforms to improve the ability of the ANZ food standards programme to protect public health. The specification and measurement of the criteria have been adapted to reflect a public health protective orientation rather than the clinical care setting to which the Donabedian model has conventionally been applied. In this orientation the criteria are:

1. Structure. Structure in the Donabedian model refers to the factors that influence the context; in this case it refers to the design of and resources used in the food standards programme. It is characterised by the governance arrangements within which the public health protective care of the programme is implemented and the membership of committees who make decisions.
2. Process. Process in the Donabedian model refers to what is being done; in this case it refers to how the performance of the food standards programme is monitored and adjusted. It is characterised by decision-making tools; public engagement; and transparency.
3. Outcomes. Outcomes in the Donabedian model refer to changes as a result of structure and outcomes; in this case, it refers to the consequences attributable to the structure and process of the food standards programme, most commonly food standards.

Food standards impact on the food supply profile, which can then influence dietary intake and nutritional health, as indicated in Fig. 1 by the dashed line to those impacts. All three impacts can also be influenced by many external factors indicated by the dashed boxes in Fig. 1. These external factors include:

∙ non-government policies and standards (e.g. a retailer’s quality standards);
∙ other government food policies and regulation (e.g. food taxes);
∙ supply-chain factors (e.g. drought conditions), food marketing, promotion and retail (e.g. food advertising);
∙ an individual’s knowledge, attitudes and personal preferences (e.g. vegetarian preferences);
∙ the genetic profile of consumers; and
∙ non-dietary lifestyle factors (e.g. physical inactivity).

### Data collection

#### Structure and process criteria

##### Governance, membership of decision-making committees, decision-making tools and transparency of the Australia and New Zealand food standards programme

Data on governance, membership of decision-making committees, decision-making tools and transparency of the ANZ food regulation system were collected from a Ministerial policy statement^(^ ^18^ ^)^, food regulation governance documents^(^ ^19^ ^–^ ^22^ ^)^ and FSANZ strategic plans, procedural documents and website^(^ ^13^ ^,^ ^23^ ^–^ ^25^ ^)^ attempting to clarify what the protection of public health means and how it should be applied in relation to food standards practice.

##### Public engagement

Public engagement principles in developing standards are outlined on the FSANZ stakeholder engagement webpage^(^ ^26^ ^)^. For instance, formal meetings are conducted to engage with: consumers and public health professionals; industry; and domestic and international government agencies. The webpage also explains that any member of the public can make an application to vary the Food Standards Code (the ‘Code’ hereafter). Data on what is occurring in practice in public engagement were collected by examining summary documents that were used to identify applications and proposals to amend the Code submitted between 1991 and 2016 (the most current data available at 14 February 2018): Finalised Proposals (as at 1 September 2016)^(^ ^27^ ^)^ and Finalised Applications (as at 1 September 2016)^(^ ^28^ ^)^. Information in these documents was supplemented by examining two online databases: FSANZ database of proposals^(^ ^29^ ^)^ and FSANZ database of applications^(^ ^30^ ^)^. Identification and analysis of the result of each application (withdrawn, rejected, gazetted) were beyond the scope of the current project.

For each application and proposal, the application and proposal name and the name of the applicant and proposer were entered into an Excel spreadsheet and were categorised into the following stakeholder groups: FSANZ (proposer); government agency/department; public health agency/individual; industry agency/individual; academic/researcher; consumer group/individual consumer; unclear (applicant name provided, but unable to be classified); and applicant data not available.

#### Outcome criteria

The food standards relating to voluntary food fortification (Standard 1.3.2), nutrition, health and related claims (Standard 1.2.7) and the nutrition information panel (NIP; Standard 1.2.8) were selected as indicators of the food standards characteristic of the outcome criteria outlined in Fig. 1 because they can be powerful interventions for protecting public health by increasing the population’s exposure to a nutrient(s) and informing consumers about some aspects of the nutritional quality of foods.

Data were collected on the food standards’ details in relation to how their provisions matched the recommendations of the Australian Dietary Guidelines. For example, categorising foods eligible to be fortified and/or displaying nutrition, health and related claims and then classifying them as either a ‘five food group’ food or a discretionary food.

#### Impact on food supply profile

The profiles of ‘five food group’ foods and discretionary foods in the marketplace were the indicators of the impact of food standards on the food supply profile. As there are no publicly available data on the number and types of products or their prevalence within the total ANZ food supply, in August 2017 we conducted a rapid literature review of studies reporting the profile of food products fortified and/or displaying nutrition, health and related claims. After searching this preliminary database, we examined PubMed using the following search query: ((‘nutrition claim’ (tiab) OR ‘nutrition claims’ (tiab) OR ‘health claim’ (tiab) OR ‘health claims’ (tiab) OR ‘nutrient claim’ (tiab) OR ‘nutrient claims’ (tiab) OR fortify (tiab) OR fortified (tiab) OR fortification (tiab) OR ‘food label’ (tiab) OR ‘food labels’ (tiab) OR ‘food labelling’ (tiab))) AND (Australia (tiab) OR ‘New Zealand’ (tiab) OR FSANZ (tiab) OR ‘food standard’ (tiab) OR ‘food standards’ (tiab)).

Search results were screened by title, abstract and then full text. The reference lists of included articles were hand-searched. Relevant references were retrieved and screened. Papers that analysed the foods for which nutrition, health and related claims were made in Australia and New Zealand were eligible for inclusion. Relevant results from the abstracts of included studies were extracted.

### Data analysis

The data were analysed in three steps. First, each of the criteria in Donabedian’s model was assessed for its impact and how well the ANZ food standards programme protected public health. This step involved combining the assessments of the individual characteristics within that criterion. Second, this impact assessment for each individual criterion was analysed to identify associations among the criteria and what this might mean for the way the food standards programme protected public health when considered as a whole. Third, the opportunities and necessary reforms that emerged from the assessments were identified.

## Results

The ANZ food standards programme’s structure, process and outcome criteria operate within the wider ANZ food regulation system, with the following actors being analysed in the present study:

∙ Ministerial Forum on Food Regulation (Forum), which is responsible for food regulation policy guidance and decides approval, review or rejection of draft standards^(^ ^19^ ^)^.
∙ Food Regulation Standing Committee (FRSC), which provides policy advice to the Forum^(^ ^19^ ^)^.
∙ FSANZ Board, which, broadly, ensures food standards are developed and implemented in accordance with the objectives of the FSANZ Act 1991 and ensures public-sector governance arrangements are in place to enhance confidence in FSANZ, its decisions and actions^(^ ^31^ ^)^.
∙ FSANZ, which is an independent statutory authority with responsibility for developing evidence-based food standards that satisfy the three primary objectives of Section 18 of the FSANZ Act 1991. In descending priority order, these are: (i) the protection of public health and safety; (ii) the provision of adequate information relating to food to enable consumers to make informed choices; and (iii) the prevention of misleading or deceptive conduct^(^ ^17^ ^)^. FSANZ does not have an explicit role in influencing or setting policy for food standards; or approving, implementing and enforcing food standards.

### Structure

#### Governance

The ANZ food standards programme’s governance operates to the Council of Australian Government’s (COAG) ‘Principles of best practice regulation’^(^ ^32^ ^,^ ^33^ ^)^. These Principles require efficiency in use of regulation; minimising the impact on competition; ensuring compatibility with international standards; and not restricting international or interstate trade^(^ ^34^ ^)^. The Principles are themselves shaped by two particular contexts. First, the rapid expansion of the global food supply chain and food’s parallel role as a commodity in international trade has resulted in free trade agreements among countries exerting a powerful influence over domestic food policy and regulation. Second, the modern ANZ food regulation system emerged within the micro-economic reform agenda of the late 1980s^(^ ^35^ ^)^. Since then, ongoing reforms to the ANZ food standards programme have involved changes to the structures and processes from a neoliberal approach to governance, characterised by the pursuit of deregulation and expectations that government should operate within ongoing efficiency dividends^(^ ^36^ ^)^. For example, in a 2014 speech to the House of Representatives the then Prime Minster stated, ‘Cutting red tape is at the heart of this government’s mission’^(^ ^37^ ^)^.

The COAG Principles of best practice regulation have a profound influence on roles for agencies across all areas of government. In relation to FSANZ, the Principles require a case to be established for setting or varying a food standard before tackling any public health problem. The case must demonstrate that the benefits to the community as a whole outweigh the costs. This principle is embedded within the FSANZ Act 1991^(^ ^17^ ^)^ which specifies that FSANZ must conduct a cost–benefit analysis when assessing an application. The government’s Office of Best Practice Regulation will determine if FSANZ must also prepare a regulation impact statement using quantitative approaches to assess a standard’s economic and competition impacts, in order to assist decision makers decide whether the standard is necessary.

FSANZ must also have regard to thirteen ministerial policy guidelines^(^ ^38^ ^)^ when setting or varying food standards. In particular, the following two policy guidelines influence the ability of food standards to protect public health in the context of dietary risk factors.

##### Policy guideline for the fortification of food with vitamins and minerals

Policy guidance on the development of permissions for the addition of vitamins and minerals to food^(^ ^39^ ^)^ is informed by ‘High order’ and ‘Specific order’ policy principles. Higher order policy principles include that the development of standards regulating the addition of vitamins and minerals to food must have regard to the objectives established in the FSANZ Act 1991. Specific order policy principles exist separately for mandatory and voluntary fortification. An example of a specific order policy principle for the mandatory addition of vitamins and minerals to food is that it should be, ‘required only in response to demonstrated significant population health need taking into account both the severity and the prevalence of the health problem to be addressed’. As an example of a specific order policy principle for the voluntary addition of vitamins and minerals to food, permission is only to be given where, ‘there is a need for increasing the intake of a vitamin or mineral in one or more population groups demonstrated by actual clinical or subclinical evidence of deficiency or by data indicating low levels of intake’.

##### Policy guideline for nutrition, health and related claims

The policy guidance^(^ ^40^ ^)^ defines nutrition, health and related claims as including all claims referring to nutrient content, nutrient function, enhanced function, reduction of disease risk or maintenance of normal health. Claims meeting set nutrient composition criteria can be made as nutrition content claims which refer to the nutrient content of a food, e.g. ‘source of calcium’, or general-level health claims which refer to a nutrient in a food or the food itself, and its effect on health, e.g. ‘calcium is important for building strong bones and teeth’. Manufacturers are permitted to self-substantiate a food–health relationship based on a systematic literature review and, following an administrative process of notification to FSANZ, a general-level health claim may be made on the food label or in food advertising. A high-level health claim refers to a nutrient in a food and its relationship to a serious disease or to a biomarker, e.g. ‘diets high in calcium may reduce the risk of osteoporosis’. High-level health claims are permissible when a pre-approved food–health relationship has been established by a systematic literature review. Additional criteria and conditions regarding the use of nutrition, health and related claims on foods are specified in the policy; for example, they are not permitted to be displayed on alcoholic beverages.

#### Membership of decision-making committees

Currently (late 2018), the FSANZ Board is comprised of twelve members with expertise and experience related to the following sectors: government (*n* 2), food safety (*n* 2), public health and nutrition (*n* 2), food science and technology (*n* 2), consumer affairs (*n* 1), food industry (*n* 2) and hospitality (*n* 1)^(^ ^22^ ^)^. At this same period the Forum and the FRSC have fifteen and twenty-one members, respectively, from the following government departments: Health and/or Food Safety (*n* 10 and 12, respectively); Agriculture/Primary Industries (*n* 4 and 8, respectively); and the Local Government Association (*n* 1 for each)^(^ ^41^ ^,^ ^42^ ^)^. For the Forum, each jurisdiction has one vote and one Minister takes the lead; currently the Health Ministers in all jurisdictions lead except in New South Wales where the Primary Industries Minister leads. However, the lead Minister must represent a ‘whole-of-jurisdiction’ view incorporating perspectives of health, agriculture and other relevant departments. The FRSC members are Senior Executives from the jurisdictions represented on the Forum and are also required to reflect a whole-of-jurisdiction view and have the authority to make decisions on behalf of their jurisdictions.

### Process

#### Decision-making tools

The protection of public health when setting ANZ food fortification, nutrition, health and related claims standards is informed by science-based tools to assess the benefits and risks of those standards. The primary tools to assess public health benefits are literature review and dietary modelling. For assessing, managing and communicating public health risk, the primary tool is the risk analysis framework. In addition, the nutrient profiling scoring criterion (NPSC) is used as a risk management tool in the context of determining the eligibility or ineligibility of specific food products to be voluntarily fortified and/or display health claims.

##### Literature review

The literature review procedure is used to assess the public health need for and benefit of fortification as well as the evidence to substantiate general- or high-level health claims. FSANZ uses well-established procedures for designing and implementing literature reviews^(^ ^24^ ^)^.

##### Dietary modelling

Dietary modelling is used as a tool to assess the potential public health benefit of fortification through its ability to estimate the additional intake of an existing ‘limited’ nutrient under various fortification scenarios.

##### Risk analysis framework

**Fig. 2:**
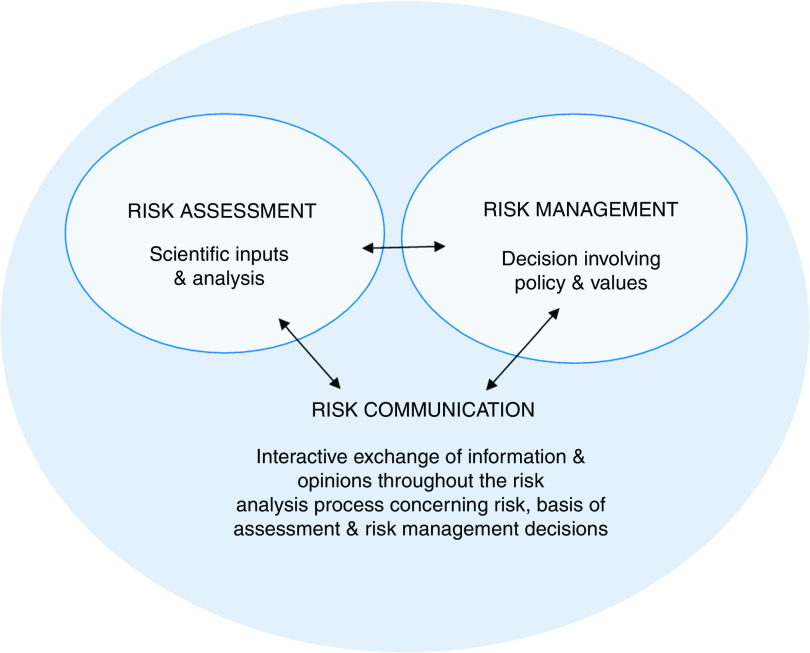
(colour online) The Food Standards Australia New Zealand (FSANZ) risk analysis framework. Adapted from FSANZ^(^ ^25^ ^)^ andWHO/FAO^(^ ^43^ ^)^

A risk analysis framework is used to analyse risk from an application or proposals to vary the Code. Historically, the ANZ food standards programme used Codex procedures and assessed risk primarily in terms of microbial/toxicological concerns. Then in 2009 Codex extended its risk analysis framework to incorporate a nutrition perspective, framing nutrition risk in terms of adverse health effects from inadequate and/or excessive intakes of nutrients^(^ ^4^ ^)^. Subsequently, FSANZ has based its nutrition risk analysis on the Codex’s procedures, prefacing each Codex risk assessment step with ‘nutrient related’^(^ ^25^ ^)^. According to FSANZ ‘nutritional risk’ is, ‘The likelihood and severity of an adverse effect from an inadequate or excessive intake of a nutrient-related hazard’^(^ ^25^ ^)^. FSANZ has developed sophisticated dietary modelling procedures to assess the risk associated with food fortification in relation to: inadequate or excessive nutrient intake; the consumption of the food vehicle; and the dietary pattern surrounding that food vehicle. Figure 2 illustrates the generic risk analysis framework upon which the FSANZ risk analysis framework is based, with its three interlinked components^(^ ^25^ ^,^ ^43^ ^)^:

1. risk assessment, a scientifically based process consisting of the steps of (i) hazard identification, (ii) hazard characterization, (iii) exposure (intake) assessment and (iv) risk characterization;
2. risk management, the process of considering the findings of the risk assessment and the views of interested parties to weigh up policy options; and
3. risk communication, the interactive exchange of information and opinions with interested parties throughout the risk analysis process.

The risk analysis framework is used to assess the risk of inadequate or excessive nutrient intake, relative to nutrient reference standards, that would result from setting food standards for voluntary and mandatory fortification. A dietary modelling procedure is used to assess nutrient intake, taking into account which food vehicle is fortified and the level of nutrient addition. The nutrient intake assessment is undertaken on a case-by-case basis varying with the role in the diet of the food vehicle to be fortified. For example, when FSANZ conducted a risk assessment on mandating the use of iodised salt in bread, it based its modelling specifically on bread consumption and the impact this would have on total iodine intake.

##### Nutrient profiling scoring criterion

The NPSC is used to assess whether or not a food is eligible to be voluntarily fortified and/or display a health claim. The NPSC is built around an algorithm that calculates a nutrient profile score for a food based on a combination of: (i) baseline points, which are themselves based on the content of energy, saturated fat, total sugar and sodium per 100 g (or ml); (ii) fruit and vegetable points; and sometimes (iii) protein points and/or fibre points. Foods exceeding a reference score are ineligible for voluntary fortification, or to display general- or high-level health claims. The detailed information for calculating a nutrient profiling score is set out in the *Australia New Zealand Food Standards Code – Schedule 5 – Nutrient Profiling Scoring Method* ^(^ ^44^ ^)^.

#### Public engagement

Among stakeholders applying for or proposing changes to food standards, a total of 1031 applications and proposals was identified, with 406 excluded because the applicant’s name could not be identified from the documentation available online. This left 625 applications and proposals. Table 1 indicates that the majority of applications were from industry agencies (*n* 237, 38 %) and FSANZ made a relatively large number of proposals (*n* 315, 50 %). Of the sixty-four applications submitted by government agencies, fifty-three were in relation to Maximum Residue Limits. Twenty-three applications and proposals were explicitly about nutrition, health and related claims, with most originating from FSANZ (*n* 22, 96 %). Eighteen applications and proposals were explicitly about food fortification and the majority of those were submitted by industry (*n* 14, 78 %).

**Table 1:** Number of applications and proposals, by stakeholder group, among stakeholders applying for or proposing changes to food standards in Australia and New Zealand between 1991 and 2016 (the most current data available at 14 February 2018)

	Applications/proposals	
Stakeholder group	*n*	%
Food Standards Australia New Zealand (FSANZ)	315	50
Government agency or department	64	10
Public health agency or individual	1	<1
Industry agency or individual	237	40
Academic or researcher	0	0
Consumer group or individual consumer	4	<1
Unclear (applicant name provided, but unable to be classified)	4	<1
Total	625	100

#### Transparency

Transparency in the treatment of information and deliberations varies across the three governance levels of the ANZ food regulation system. All documents prepared for the Forum are treated as sensitive, unless otherwise agreed by the Forum, and distributed only on a strict ‘need-to-know’ basis. The same process applies to the FRSC; confidential information and agenda papers, draft minutes, action lists and endorsed minutes are not released for public access. Instead, a public communiqué is issued after each Forum meeting. By contrast, FSANZ has a public register of applications and proposals as well as related information being made available on its website for communication with and scrutiny by stakeholders.

### Outcomes

#### Food Standard 1.3.2: Vitamins and minerals

In accordance with policy guidance for food fortification, Standard 1.3.2^(^ ^45^ ^)^ regulates the voluntary addition of vitamins and minerals to general purpose foods. There is no publicly available record at FSANZ or the broader ANZ food regulation system that details the types and numbers of voluntarily fortified foods available in the marketplace.

#### Food Standard 1.2.7: Nutrition, health and related claims

In accordance with policy guidance for nutrition, health and related claims, Standard 1.2.7^(^ ^46^ ^)^ sets out the requirements which permit food businesses to voluntarily make claims on food labels and in advertising. Food businesses can base general-level health claims on one of the more than 200 pre-approved food–health relationships in the Standard or self-substantiate a food–health relationship in accordance with requirements set out in the Standard. Thirteen pre-approved high-level health claims can currently be made. There is no publicly available record in the ANZ food regulation system that details the types and numbers of food products in the marketplace that display nutrition, health and related claims.

#### Food Standard 1.2.8: Nutrition information requirements

Since 2000, labelling Standard 1.2.8^(^ ^47^ ^)^ requires most food labels to provide a NIP with basic information per 100 g (or ml) and serving size (with details about the number of servings in the package). The information that the NIP mandates includes: energy; protein; fat – total; fat – saturated; carbohydrate – total; carbohydrate – sugars (but not ‘added’ or ‘free’ sugar); sodium; and any other nutrient or biologically active substance for which any claim is made.

### Impact on food supply profile

Six papers met the rapid review inclusion criteria. These papers demonstrated the extent to which food standards for fortification and nutrition, health and related claims are being used by manufacturers of discretionary foods sold in Australia or New Zealand. The two main findings from the review are that: (i) nutrition, health and related claims based on nutrient profiling criteria are mostly on ‘healthy’ food products but are also on one-third of ‘less-healthy’ products in New Zealand supermarkets^(^ ^48^ ^)^ and these findings are broadly similar to another study previously conducted in New Zealand supermarkets^(^ ^49^ ^)^; and (ii) two Australian studies^(^ ^50^ ^,^ ^51^ ^)^ reported that a substantial number of discretionary foods carried nutrition, health and related claims. The second of these reported that the highest proportion of these claims was on sports drinks, energy drinks, sports bars and breakfast cereals^(^ ^51^ ^)^.

## Discussion

### Assessment of the public health protection performance of the Australia and New Zealand food standards programme

#### Structure

Structurally the ANZ food regulation system’s separation of its food regulation policy making (Forum) and science/standards setting (FSANZ) arms presents challenges to the food standards programme’s standards setting role. This separation distances evidence inputs from policy-making processes. While FSANZ has world-class nutrition science expertise, the broader ANZ food regulation system structure has less direct access to such expertise to inform decisions. Over the last 10 years, nutrition capacity, expertise and experience have been reduced in many government departments as nutrition scientists’ positions are removed or reassigned. For example, following the 2010 disbandment of the Strategic Intergovernmental Nutrition Alliance there is no nationally coordinated nutrition advice within state and territory governments^(^ ^52^ ^)^. This means that expert advice for the FRSC and the Forum is not always immediately available when considering applications and proposals to vary food standards. Also, whereas the existence of the FSANZ public register and website provides reassurance in the transparency of food standards setting activities, those activities operate within the broader ANZ food regulation system where policy agendas are set and yet have less transparency and scope for public participation. One promising initiative to help tackle this concern has been the FRSC’s trialling of a ‘stakeholder roundtable’ approach to improve stakeholder awareness of policy activities within the food regulatory system^(^ ^21^ ^)^.

The absence of an explicit FSANZ role to evaluate the implementation and impact of food standards in relation to type and number of foods that are fortified and/or displaying nutrition, health and related claims diminishes its capacity to protect public health. Without this information, there is less capacity for accountability of past decisions and a missed opportunity to get evidence that could inform future decisions.

The legislated requirement to prepare a cost–benefit analysis and a regulation impact statement where needed challenges the ability to proactively set food standards to protect against dietary risk factors.

Challenges to proactively setting food standards to protect against dietary risk factors arise at both the policy setting and standards setting levels of the ANZ food regulatory system structure. On the Forum, the mix of Health and Agriculture Ministers and representation from New Zealand and the consensus decision-making process can present challenges in taking progressive decisions to tackle public health nutrition problems. At the standards setting level the legislated requirement to prepare a cost–benefit analysis and a regulation impact statement challenges the ability to proactively set food standards to protect against dietary risk factors. There is no set formula for conducting a cost–benefit analysis or defining precisely what ‘counts’ as a cost and as a benefit. It is relatively straightforward to estimate the cost to a food manufacturer of complying with a standard, such as changing a food label. But it is more challenging to estimate the public health benefits attributable to that label change when benefits might accrue from multiple inputs and take years to take effect.

These structural concerns are not peculiar to the ANZ food standards programme; indeed more serious concerns have been raised elsewhere. Lobstein comments that the UK Food Standards Agency is ‘far too cosy with big business: even the government seems to think it is no longer serving consumers’ interests’^(^ ^53^ ^)^. This relationship with vested interests highlights the importance of strong governance to build trust in that Agency and avoid risk of regulatory capture^(^ ^54^ ^)^. A non-government organisation has expressed a similar sentiment when commenting on the need for Codex to be protected from commercial influence^(^ ^55^ ^)^. It has been reported that conflicts of interest are embedded in the structures and processes of the US Food and Drug Administration and statutory amendments are needed to ‘rebalance’ the composition of advisory committees and the scientific basis for informing dietary recommendations^(^ ^56^ ^)^. Devaney has argued that the Irish food regulation system has limited awareness of and engagement with the public and it needs to increase its accountability, transparency and effectiveness^(^ ^57^ ^)^.

#### Process

The processes of the ANZ food standards programme assess and manage risks in terms of inadequate and excess nutrient intakes as well as excessive and unbalanced dietary intakes, e.g. by profiling whether a food is aligned with the Australian Dietary Guidelines before determining if it is eligible to display nutrition, health and related claims. The risk analysis framework is a particularly effective decision-making tool in a fortification context when setting nutrient levels permitted to be added to foods to avoid inadequate/excessive nutrient intake. However, in their current forms the risk analysis framework and its NPSC are not sufficiently aligned with contemporary understandings of nutrition science to adequately protect public health from dietary excesses and imbalances when setting food standards. This is because the designs of both are predicated on a reductionist approach to risk assessment, i.e. specifying and measuring risk more in terms of impact on intake of an individual nutrient than on intake of a food or a dietary pattern.

The Australian Dietary Guidelines explain that the principal risks for obesity and NCD are not individual nutrients, but dietary patterns characterised by an inadequate intake of ‘five food group’ foods and an excessive intake of discretionary foods^(^ ^58^ ^–^ ^60^ ^)^. The nutrient-oriented approaches of the risk analysis framework and the NPSC isolate nutrients from foods, making it a challenge to determine whether the source of the nutrient was a ‘five food group’ food or a discretionary food. As Mozaffarian comments, not all kilojoules (and nutrients) are created equal in terms of obesity and NCD risk, because it depends on the source foods within which they are found^(^ ^61^ ^)^.

The present study’s findings highlight that substantially more applications to the ANZ food standards programme come from commercial rather than public health interests. The public health sector’s engagement with the Programme appears to be oriented mostly towards reacting to applications from food manufacturers, with the sector attempting to protect public health rather than proactively submitting applications to promote public health. There is also a differential capacity between commercial and public health interests to engage with the Programme^(^ ^62^ ^)^. Transnational food companies usually have greater capacity than public health organisations and practitioners to advocate for their interests and influence food policy decision making in Australia^(^ ^63^ ^–^ ^65^ ^)^ and internationally^(^ ^66^ ^)^. Researchers report that the private sector is increasingly influencing regulatory decision-making structures and processes intended to protect public health, a phenomenon referred to as ‘corporate capture’ of regulators^(^ ^67^ ^)^.

Many of these procedural characteristics have been seen in other food standards programmes. Participation in the Joint FAO/WHO Food Standards Programme has led to the Codex risk analysis framework being commonly used. However, this frames risk primarily in terms of inadequate or excessive nutrient intake, rather than in terms of food consumption and risk of dietary excess or imbalance. Codex officials have commented that over its 50 years of existence Codex has delivered a strong public health protection performance in managing and mitigating chemical, microbiological and nutritional food safety risks of consumers^(^ ^68^ ^)^. Codex is less forthcoming about its public health protection performance in the context of managing and mitigating dietary excess and imbalance risks.

#### Outcomes

‘Are we really fixing up the food supply?’ is a question the leading nutrition policy scientist Joan Gussow posed around the time of the inception of the modern ANZ food standards programme^(^ ^69^ ^)^. Over the period of its existence it been responsible for the setting of food standards that have an enviable reputation for their effectiveness in helping protect the population from inadequate and excessive nutrient intake and assisting consumers make informed food choices, e.g. the mandating of the NIP.

The findings from the present study also show that over the same period the ANZ food standards programme has overseen the setting of food standards with liberal provisions for the use of voluntary fortification and nutrition, health and related claims. However, the impact of these standards has not always been consistent with the Australian Dietary Guidelines’ recommendations. Whereas these standards often are poorly accessible to whole ‘five food group’ foods because physically it can be difficult to fortify these foods and/or have a label on which to display claims, they are highly accessible to discretionary foods and have coincided with their proliferation and marketing^(^ ^70^ ^,^ ^71^ ^)^.

Similar outcome characteristics to those observed for the ANZ food standards programme – in terms of fortification and nutrition, health and related claims standards – have been reported for other national food standards programmes. Worryingly, there also has been a burgeoning number and reach of ‘ultra-processed’ food products, i.e. industrially formulated foods that contain few whole food components, in countries around the world^(^ ^72^ ^–^ ^81^ ^)^. This is a concern because of the emerging evidence base indicating a positive association between consumption of ultra-processed food products and certain adverse health outcomes^(^ ^82^ ^,^ ^83^ ^)^.

A synthesis assessment of these observed ANZ food standards programme dimensions that align with each of the Donabedian model criteria (structure, process and outcome) indicates an overall mixed performance in terms of the quality of public health protection. The Programme sets evidence-informed standards mandating food fortification and nutrition information on labels that perform well in protecting the public against inadequate or excessive nutrient intake. Conversely, the Programme performs less well in public health protection in relation to helping protect against dietary risk factors for obesity and NCD.

The Donabedian model illustrates that an association exists between the structure, process and outcome dimensions of the ANZ food standards programme and its public health protection performance. For instance, food standards related to voluntary fortification and nutrition, health and related claims are coinciding with the proliferation and marketing of discretionary foods. This association arises from shortcomings in the decision-making tools for assessing public health benefits and risks of those food standards, and these in turn are related to shortcomings with policy guidance for food fortification and nutrition, health and related claims.

### Opportunities for food standards programmes to protect public health against dietary risk factors for obesity and non-communicable diseases

To protect public health against dietary risk factors for obesity and NCD and accord with dietary guidelines, food standards programmes can provide two opportunities. First, set food standards to proactively promote a healthy food environment by promoting ‘five food group’ foods. Second, set food standards to reactively protect against an unhealthy food environment by avoiding inadvertent promotion of discretionary foods. An example of a food composition and a food labelling standard for each of these food standards types follows.

1. Proactively set food standards to promote a healthy food environment:
  a. Mandate the disclosure of the amount of added (free) sugar on the NIP where added sugar is an ingredient to both ‘five food group’ and discretionary foods.
  b. Mandate front-of-pack labelling in the form of positive symbols for ‘five food group’ foods and warning symbols for discretionary foods.
2. Reactively set food standards to protect against an unhealthy food environment:
  a. A food composition standard that explicitly excludes discretionary foods from being eligible for voluntary fortification permissions. For instance, Section 3.3.1 of the Codex Alimentarius’ *General Principles for the Addition of Essential Nutrients to Foods* states, ‘The selection of foods to which essential nutrients may be added should be in line with the intended purposes of nutrient addition …, dietary patterns, socioeconomic situations and the need to avoid any risks to health’^(^ ^84^ ^)^.
  b. A food labelling standard that explicitly excludes discretionary foods from being eligible for nutrition, health and related claims.

### Policy implications: reforms to improve public health protection performance of food standards programmes

The current research aimed to not only assess performance but also identify reforms necessary for positioning a food standards programme to help protect public health against dietary risk factors. The aim is particularly relevant in the context of the Forum’s identification of supporting ‘public health objectives to reduce chronic disease related to overweight and obesity’ as one of three priority areas for the food regulatory system 2017–2021^(^ ^14^ ^)^. In this section reforms are identified against each of the three criteria of the Donabedian model.

#### Structural reforms

##### Governance

Policy leadership is needed to put in place a national nutrition policy framework that among other benefits will help align ANZ food regulation system policies and ANZ food standards programme standards with preventing obesity and NCD. A similar type of joined-up policy approach has been called for in the USA to address the fragmentation and inconsistent messages across the food regulation system and food standards programme for food safety regulation in that country^(^ ^85^ ^)^.

The regulation impact statement scope needs to adopt a more relevant specification and measurement of the direct and indirect economic and social costs associated with the burden of obesity and NCD and the benefits from their prevention, when assessing food standards aimed at helping protect the public from dietary risk factors.

##### Membership of decision-making committees

Membership of ANZ food regulation system decision-making committees is a matter for the relevant jurisdictions, however there is a need to constantly emphasise that the system’s primary objective is protecting public health. All decisions should adequately represent public health interests in decision making.

#### Process reforms

Critical to the process reforms is the need for a more authentic use of nutrition science to specify and assess risks when informing food standards^(^ ^86^ ^,^ ^87^ ^)^. This means that whereas a nutrient-oriented paradigm to nutrition science is relevant for analysing nutrient risks, a food/diet-oriented paradigm to nutrition science is more relevant for analysing dietary risks.

##### Reform the risk analysis framework and nutrient profiling scoring criterion

In the context of fortifying a food, the nutrient-based risk analysis framework is well designed for assessing risks associated with nutrient inadequacy or excess. However, the same risk analysis framework contradicts nutrition science associated with the recommendations of food-based dietary guidelines. In that context, assessing dietary excess and imbalance means combating inadequate dietary intake of ‘five food group’ foods and excessive dietary intake of discretionary foods. The risk analysis framework for specifying and assessing public health risk associated with food fortification needs to be reformed to capture Australian Dietary Guidelines’ recommendations.

The NPSC is a limited approach to assess public health risk to determine the eligibility of foods to display health claims. A small number of nutrients has been included in the NPSC and the ‘cut off’ levels for scoring these nutrients have been arbitrarily set. There is a lack of evidence that the ANZ food standards programme’s NPSC is predictive of health outcomes or is able to discriminate between foods based on dietary guideline recommendations; instead, it may be compromising the precautionary principle of ‘first do no harm’. The NPSC for specifying and assessing public health risk associated with health claims needs to be reformed to capture the Australian Dietary Guidelines’ recommendations.

Reform of the decision-making tools requires extending the modelling and profiling used in the risk analysis framework and NPSC, respectively, from their current nutrient (reductionist) paradigm to be inclusive of a food/diet (holistic) paradigm. Such reforms will require translating food-based dietary guideline recommendations into a form that food standards can use for individual foods or food categories. For example, a risk analysis framework or NPSC might need an added dimension to demarcate a candidate food for fortification of a nutrition, health and related claim as a ‘five food group’ or discretionary food.

##### Reform transparency

Reforms are needed to combine policy setting and standards setting responsibilities and strengthen transparency in decision making across the ANZ food regulation system. Currently FSANZ has a consultation process and public register, but the FRSC trialling of stakeholder roundtables^(^ ^21^ ^)^ notwithstanding, no equivalent is in place for the Forum and FRSC.

##### Reform processes to enable public health practitioners more equitable access to the Australia and New Zealand food standards programme decision-making committees and submission processes

Public health interest groups and individuals need to be more critically aware of and engaged with the ANZ food standards programme to help promote reforms to improve its public health protection performance. Prioritising food regulation literacy as a core competency in training programmes is one practical way to strengthen practitioners’ ability to identify and respond to the Programme’s activities^(^ ^88^ ^,^ ^89^ ^)^. Financial support for public health interests to participate in meetings is another reform that would strengthen public health engagement with the ANZ food standards programme. Consumers International has noted that consumer involvement in Codex faces challenges with lack of funding, poor transparency of Codex procedures and greater representation of industry interests over consumer interests^(^ ^90^ ^)^.

### Strength and limitations of the present research

The nature and scope of the present research is unique in that the critical analysis was conducted on the overarching ANZ food standards programme and not on its individual components in isolation. Research at this level is important because it enables the investigation to analyse the dynamics of the ANZ food standards programme’s structures, processes and outcomes as a coherent whole as well as the multiple complex interactions among the individual components. In this regard the Donabedian model was particularly well suited to the research because it provided a systematic approach for assessing the performance of the otherwise complex operation of the ANZ food standards programme. Also, its internal logic provided an analytical road map to help identify how and why problems have arisen by looking at the structures and then the processes that are responsible for outcomes.

However, the identified limited transparency with the ANZ food standards programme processes means that it is possible that not all data were located or indeed even exist to help understand and explain the dynamic processes of the Programme. In this situation the data collection process was as thorough and comprehensive as possible, as it identified and then sought out all critical official food standards programme documents related to structures and processes. The data were collected at different time points during that period and so it cannot be assumed there was a direct cause–effect relationship between individual criteria. For example, the membership profile of decision-making committees varied over time and it is not possible to link one particular profile with specific processes or outcomes. In these circumstances it was the overall profile and direction of decision making that were analysed. It is conceivable there were confounding factors that might provide alternative explanations for the food standards that emerged.

The findings presented in the current study are drawn from critical analysis of the structures, processes and outcomes in the Australasian context and so cannot necessarily be used to draw direct parallels for food standards programmes in other countries or Codex. Nevertheless, the literature does indicate the ANZ food standards programme shares similar structural, procedural and outcome characteristics with many other national food standards programmes and Codex. Also, most food standards programmes are subject to similar governance contexts in relation to global free trade and national deregulation agendas which affect their structures and processes. Therefore, there likely are relevant lessons from the ANZ food standards programme case about performance, opportunities and necessary reforms for other national food standards programmes.

In the future, a priority activity will be to develop a nutrition and health information system in which regular food and nutrition surveys are conducted, and data are available for setting and varying food standards in a timely fashion. Accompanying this activity is the need to broaden the scope of monitoring and evaluating the impacts of food standards in accordance with dietary guideline recommendations, e.g. the profile of ‘five food group’ and discretionary foods in the marketplace using voluntary fortification and health claims. Despite FSANZ introducing in 2000 an ‘evaluation strategy’^(^ ^91^ ^)^ there has been no evaluation of voluntary fortification and health claims since 2005. Currently no completed studies have assessed the effect of such claims on their longer-term impact on diet or health^(^ ^92^ ^)^.

## Conclusion

Modern food standards programmes typically perform well in protecting public health from inadequate or excessive nutrient intake. Conversely, these same programmes generally perform less well in relation to protecting public health from dietary excess and imbalances associated with obesity and NCD. This less effective performance is a consequence of being agnostic towards nutritious foods while at times inadvertently facilitating the proliferation and marketing of energy-dense, nutrient-poor food products in contradiction to the recommendations of food-based dietary guidelines. The lesson is that whereas the nature of the public health threat has changed over time, the structures, processes and outcomes of food standards programmes rarely have changed commensurately. This limits the quality of their performance in responding to contemporary dietary risks.

There are opportunities to proactively set food standards to protect public health from dietary risk factors. These opportunities would be best realised when undertaken as one component within a coherent national food and nutrition policy. Expectations for these opportunities need to be kept in perspective because food standards programmes operate within a broader food regulation system, and national food standards programmes operate within a global food standards programme. The more substantive opportunity to strengthen a programme’s public health protection performance is in a reactive orientation, i.e. by applying risk analysis tools and processes more assiduously to help protect against dietary risk factors. Paradoxically, certain nutrition-based food standards such as voluntary fortification and nutrition, health and related claims have unintentionally contributed to an unhealthy food environment. This situation is instructive in highlighting that even when setting food standards with the intention to help protect public health, there is a need to ‘first do no harm’.

Reforms to structures and decision-making processes necessary to realise these opportunities need a more authentic use of nutrition science in food standards programme activities for assessing and responding to dietary excess and imbalance risks. Reforming the risk analysis framework, including its NPSC, is a priority. This will require food standards programmes’ design and application to be fit for purpose and this means extending their nutrition science orientation from a nutrient (reductionist) paradigm to be more inclusive of a food/diet (holistic) paradigm.

## Author ORCID

Mark Lawrence, 0000-0001-6899-3983. Christina Pollard, 0000-0003-4261-4601. Tarun Weeramanthri, 0000-0003-2796-7345.
